# Influence of different stimulants on wheat (*Triticum aestivum* L.) grain in relation to the germination and early growth

**DOI:** 10.1007/s00114-025-02020-z

**Published:** 2025-09-09

**Authors:** Božena Šerá, Pratik Doshi, Lubomír Věchet

**Affiliations:** 1 Department of Environmental Ecology and Landscape Management, Faculty of Natural Sciences, Comenius University Bratislava, Ilkovičova 6, 84215 Bratislava, Slovakia; 2 Crop Research Institute, Drnovská 507/73, 161 06 Prague, Czech Republic

**Keywords:** Stimulants, Wheat, Germination characteristics, Grain priming

## Abstract

Due to the growing environmental and health concerns with chemical plant stimulants, there is a growing need to find alternative sources of plant stimulants that could help the seeds germinate and sustain their growth in the global climate change scenario. The article compares various seed stimulants such as chemical compounds (benzothiadiazole, salicylic acid, glycine betaine), alcoholic extracts from commercial plant products (English oak bark, ginger spices, turmeric spices, caraway fruits) and from wild plant leaves (Japanese pagoda tree, Himalayan balsam, stinging nettle and Bohemian knotweed) and their effects on wheat seed germination and seedling characteristics. It was found that BTH had significantly lower effect on seedling characteristics such as SG3 (%), SG5 (%), R/S III, SVI I (mm) and SVI III (mg) followed by ZO on SG3 (%), SG5 (%) and GI (unit). Significantly highest R/S III was found in SJ treatment, while SVI I (mm) and SVI III (mg) characteristics were significantly enhanced by treatment with CC and RB. It seems that such plant materials could be useful in alternative agriculture for different purposes.

## Introduction

Wheat is one of the most important crops grown worldwide for consumption. According to Igrejas and Branlard (2020), approximately 40% of the world’s population consumes bread made from wheat flour. To feed an estimated world population of 9 billion by 2050, a substantial increase in wheat production is required to ensure food security (Li et al. 2020). Wheat production is still lower compared to maize and rice despite growing in a large area of land (Saeed et al. 2022). With an increasing global climate change scenario leading to a decrease in agricultural production around the world, questions about sustainable growth of wheat and other crops are emerging. These are the reasons why we chose wheat grains as a model organism for testing the stimulation of germination and initial growth.

To improve seed germination which will further improve yield, Bradford (1986) introduced the term ‘priming’ of seeds. There are different methods of seed priming such as hydro-priming, osmo-priming, matrix priming (or drum priming), hormopriming, halo priming, chemical priming, and bio-priming (Zulfiqur 2021), which partially imbibes seeds. It helps the seed achieve a ‘germinating stage’ without causing radicle projection, which further might ensure improved germination rate and uniformity (Sung et al. 2008). Priming has been shown to help different seeds/grains to improve germination and seedling establishment under stressed conditions (Chen et al. 2012). The stimulating effect can be achieved with seed priming using various stimulants. Additionally, some plant stimulants also have potential plant protection properties. Here we discuss different plant stimulants and for the sake of convenience we grouped them into chemical, commercial plant products, and wild plants (native, ornamental, and invasive species).

Common enhancing chemicals include benzothiadiazole (BTH), salicylic acid (SA), and glycine betaine (GB). BTH was synthesised and described in 1996 as a new compound that can induce systemic acquired resistance (SAR) in plants against different plant diseases (Friedrich et al. 1996). Several research has been published on the successful protection of crops/plants against different plant diseases such as *Peronospora parasitica* in crucifers (Godard et al. 1999), Crenate boomrape control in pea (Pérez-de-Luque et al. 2004), brown stem rot in soybeans (Nafie and Mazen 2008), etc. BTH is commercialised under a registered name as Bion® which has been quite significant in protecting crops against different crop diseases.

SA is a phenolic compound that is found naturally in plants. Over the years, under different stress conditions, SA is applied exogenously for seed priming to overcome biotic and abiotic stress (Deef 2007). It was found that SA inhibits the catalase enzyme, which scavenges H₂O₂, thus generating reactive oxygen species (Bahrani and Pourreza 2012). Furthermore, it was found that SA also affects nitrate/nitrite reductases and other enzyme activities involved in nitrogen metabolism without harmful effects on environment (Jain and Srivastava 1981; Negi and Prasad 2001).

GB is one of the quaternary ammonium compounds found in plant especially in chloroplast for the adjustment and protection of the thylakoid membrane, thereby maintaining photosynthetic efficiency (Genard et al. 1991). GB occurs abundantly when the plant is under dehydration stress (Yang et al. 2003). Due to its nature of overcoming dehydration stress, GB is exogenously applied to plants that show huge negative changes in dehydration (Yang and Lu 2005). Positive effects have been compiled on the growth of different crop/plant species by exogenous application of GB under dehydration stress (Ashraf and Foolad 2007).

For the stimulation of the seeds, among the plants that are common commercial products available, the bark of English oak bark, the spices of ginger, turmeric, and caraway can be mentioned. *Quercus robur* (QR), also known as English oak, is a large, rugged, deciduous broadleaved tress that are native to Europe, western Asia, and northern Africa (Almeida et al. 2008). Oak extracts have been traditionally used for therapeutic purposes (Almeida et al. 2008). A chromatographic analysis of oak showed that the extracts contain several polyphenols and volatile compounds such as gallic acid, 5-(hydroxymethyl) furfural, 2-furanoic acid, aesculetin, vanillic acid, vanillic aldehyde, syringic acid, syringaldehyde, scopoletin, ferulic acid, coniferaldehyde, sinapaldehyde, ellagic acid and its derivatives and derivatives of gallic acid (Cadahía et al. 2001). These different polyphenols and other volatile compounds found in oak leaf litter when leached in the soil are found to influence the structure of the ectomycorrhizal community (Garnett et al. 2004).

*Zingiber officinale* (ZO) is a perennial Herbaceous plant growing up to 1 m. It is available in fresh, dried, or extract form and demand for it is increasing worldwide (Mbaveng and Kuete 2017). ZO, commonly called ginger, has been used for medicinal purposes for more than 2000 years with a range of biological properties (Dhanik et al. 2017).

*Curcuma longa* (CL), also known as turmeric, is a common spice found throughout the East and is traditionally used for cooking due to its aroma and taste (Govindarajan and Stahl 1980). Due to its yellow colour, it is called “the golden spice” and is used for more than 4000 years for its medicinal purposes (Prasad and Aggarwal 2011). The product turmeric is made after processing the turmeric rhizome (Prasad and Aggarwal 2011). Several compounds are found in turmeric, such as curcuminoids consist of curcumin demethoxycurcumin, 5′-methoxycurcumin, and dihydrocurcumin; which are found to be natural antioxidants (Ruby et al. 1995). In a standard product, turmeric contains moisture (> 9%), curcumin (5–6.6%), extraneous matter (< 0.5% by weight), mould (< 3%), and volatile oils which include d-α-phellandrene, d-sabinene, cinol, borneol, zingiberene and sesquiterpenes (< 3.5%) (Prasad and Aggarwal 2011).

*Carum carvi* (CC), commonly known as caraway, is an important medicinal plant (Seidler-Łożykowska et al. 2013). Caraway fruits are traditionally recommended to cure indigestion, pneumonia, and as a carminative, appetiser, and galactagogue (Rasooli and Allameh 2016). The chemical components of caraway fruits consist of essential oil, fatty acids (petroselinic, linoleic and oleic acids), protein, carbohydrate, phenolic acids (caffeic acids), flavonoids (quercetin, kaempferol) (Olennikov and Kashchenko 2014; Sachan et al. 2016). Caraway has different biological activities such as antimicrobial and antioxidant activity and is also used for medicinal purposes (Mahboubi 2019).

A variety of wild plants that are invasive (Himalayan balsam, Bohemian knotweed), ornamental (Japanese pagoda tree), and native (stinging nettle) and in Europe were also investigated for their stimulation effect and growth on wheat grains.

*Impatiens glandulifera* (IG) is one of the most problematic invasive plant species considered in Europe, also known as Himalayan balsam. It was intentionally introduced as an ornamental and nectar-producing plant in Europe (Pyšek and Prach 1995). The main distribution of Himalayan balsam is in the Himalayan and Eurasian regions that have a temperate climate (Janssens et al. 2006). Himalayan balsam is an erect, solitary, tall, thick stem, green to reddish in colour, usually simple or branching, usually 5 − 50 mm in diameter (Beerling and Perrins 1993). Different phenolic compounds were isolated that were flavonoids (Tanner et al. 2014; Szewczyk et al. 2019) and were studied for their antioxidant properties (Szewczyk et al. 2019).

*Reynoutria* x *bohemica* (RB) is another invasive plant species that was the result of hybridisation found in Europe, North America, and Oceania (Schuster et al. 2011). An HPLC method revealed that the phytochemical profile of RB was found to be intermediate between the two parent species, *R. japonica* and *R. sachalinensis* (Nawrot-Hadzik et al. 2018). Chemical constituents such as piceid, chlorogenic acid, epicatechin, resveratrol, catechin, quercetin-3-beta-d-glucoside were found in RB (Vrchotová et al. 2010). Allelopathic, antifungal, and antioxidant effects of *Reynoutria* spp. have also been reported (Daayf et al. 2000).

*Sophora japonica* (SJ) (Japanese pagoda tree) is an introduced species in Europe as an ornamental plant. It is a shrub species belonging to the *Fabaceae* family. The flowers and flower buds of this tree are traditionally used to treat different medical conditions (He et al. 2016). Around 153 constituents are identified including flavonoids, isoflavonoids, triterpenoids, alkaloids, mineral elements and amino acids are identified (He et al. 2016). The flavonoid glycoside rutin has been extensively studied for pharmacological effects including antioxidant, anti-inflammatory, anticancer, antidiabetic, anti-adipogenic, kidney-protective, cardioprotective, neuroprotective, antimicrobial and antiasthma effects (He et al. 2016).

*Urtica dioica* (UD), commonly known as stinging nettle, is a perennial herbaceous flowering plant, native to Eurasia (Dhouibi et al. 2020). For a long time, it has been used in alternative medicine, food, paint, fibre, manure, and cosmetics (Asgarpanah and Mohajerani 2012). Flavonoids, tanins, volatile compounds, and sterols are some of the phytochemical compounds commonly found (Krystofova et al. 2010). Stinging nettle can improve nitrogen and phosphate, promote the biodiversity of local flora and fauna (Dreyer et al. 2002).

Some of the stimulants in this investigation were tested against *Blumeria graminis* f. sp. *tritici* in winter wheat (Věchet and Šerá 2015). However, in their study the stimulation effect was not studied. This study aims to investigate the effects of diverse stimulatory treatments of varying origins on wheat (*Triticum aestivum*) seed germination and seedling growth parameters, with the objective of identifying interventions that improve seed viability, germination rate, and early seedling vigor.

## Material and methods

### Stimulant preparation

The sources for stimulants can be divided into three groups as shown in Table 1. They were used as chemical compounds: water solutions of benzothiadiazole (BTH; 1.2 mM; commercially produced as Bion and containing 50% of BTH), salicylic acid (SA; 1 mM), glycine betaine (GB; 0.3 M).

**Table 1:** Overview of used stimulants and their division into three groups

Group of stimulants	Chemical/plant source	Solution/extract	Symbol
Chemicals	Benzothiadiazole	benzothiadiazole solution	BTH
	Salicylic acid	salicylic acid solution	SA
	Glycine betaine	glycine betaine solution	GB
Commercial products	*Quercus robur* L	bark extract	QR
	*Zingiber officinale* Roscoe	spices extract	ZO
	*Curcuma longa* agg	spices extract	CL
	*Carum carvi* L	fruits extract	CC
Wild plants	*Impatiens glandulifera* Royle	leaves extract	IG
	*Reynoutria. x bohemica* Chrtek & Chrtková	leaves extract	RB
	*Sophora japonica* L	leaves extract	SJ
	*Urtica dioica* L	leaves extract	UD

The second group of stimulants contains commercial products (FA Bylík) of plant origin namely bark from *Quercus robur* (QR) commonly known as English oak, fruits of *Carum carvi* (CC) caraway seeds, *Zingiber officinale* (ZO) commonly known as ginger and *Curcuma longa* turmeric (CL) commonly known as turmeric.

The third group for stimulants were plants collected from the wild. These were samples from *S. japonica* (SJ) commonly known as Japanese pagoda tree, *Urtica dioica* (UD) commonly known as stinging needle, *Impatiens glandulifera* (IG) commonly known as himalayan balsam, and *Reynoutria* × *bohemica* (RB) commonly known as bohemian knotweed. Leaves of these wild plants were collected at the time of maturity from three different vital populations, then they were dried (62 h at 50 °C), and sample was prepared for individual species. The extracts from commercial material and from wild plants were made from 100 g of dry matter and 850 ml of 20% ethanol with intensive shaking for 5 h and subsequent filtering of the sediment. The extracts were stored in the dark at 6 °C. The grouping was done for the sole purpose of convenience and was not involved in the statistical analysis. The reason behind not comparing the data according to the groups was to investigate the effect of each treatment individually rather than collectively on wheat grain germination and grain characteristics.

### Grain treatment

Common wheat grains (*Triticum aestivum* L. cv. Kanzler) weighing 25 g were poured with 500 mL of the given treatments and gently shaken with a rotary shaker at room temperature (24 h, 20 °C). The liquid was then filtered off, and the grains were dried and stored in the dark at room temperature for two weeks (principle of seed priming). A total of 150 wheat grains were used for each type of pre-treatment. The control grain sample was not treated with any stimulant.

### Germination test

Five plastic Petri dishes (diameter 9 cm, 3 × KA quality filter paper, 6 mL of distilled water) were used as five repetitions (30 grains/Petri dish) for one treatment (150 grains/treatment). The dishes were stored in an incubator (darkness, temperature 20 °C) for 5 days. The number of germinating grains (BBCH scale at least level 05) was monitored, and a grain was considered germinating if the radicle was at least 1-mm long. On the 3rd and 5th days, the following data were determined: the number of germinated grains, the length of the seedling shoot (mm) and the largest length of the seedling roots (mm). On the last day of cultivation (5th day), the grown plants were harvested, dried and their weight/dish (g) with a laboratory scale (balance sensitivity 0.001 g) was determined after drying. The following characteristics of seed germination and seedling early growth were calculated according to the methodology (Šerá 2023): seed germination (SG, %) on the 3rd and 5th days (SG3, SG5), germination index (GI), root: shoot ratio I (R/S I), root: shoot ratio III (R/S III), seedling vitality index I (SVI I, mm), and seedling vitality index III (SVI III, mg).

### Statistical analyses

The data obtained was statistically analysed using R software (R Core Team, 2021). Only data obtained for the treatment irrespective of the group on seed germination characteristics were compared by one-way ANOVA followed by Tukey test at a 95% confidence level.

## Results

**Fig. 1:**
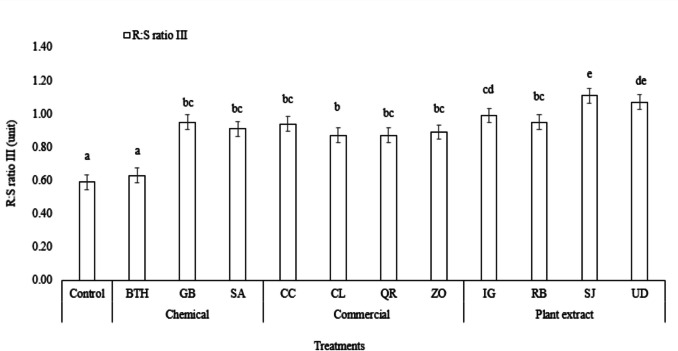
Comparison of R/S III for different treatments. Different letters on top of the error bars indicate statistically significant difference compared to control at 95% confidence level

**Fig. 2:**
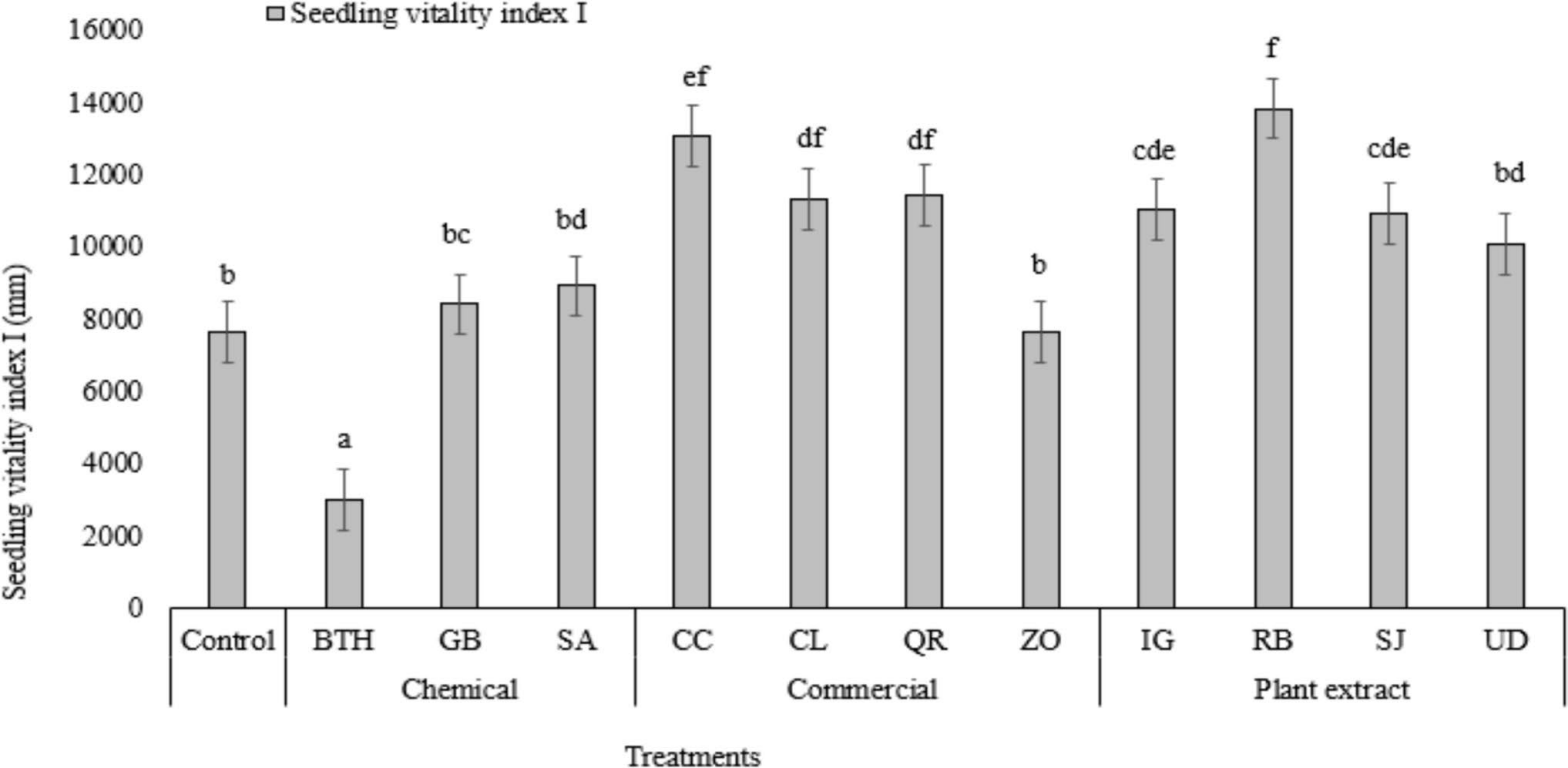
Comparison of seedling vitality index for different treatments. Different letters on top of the error bars indicate statistically significant difference compared to control at 95% confidence level

Positive increases in measured parameters at a statistically significant level will be presented first. They are the characteristics of R/S III and SVI I, when most values of the treatments were significantly higher compared to the control set (Figs. 1, 2).

As can be seen from Fig. 1, the lowest value of R/S III was observed in BTH treatment. The other chemical treatments with GB and SA, commercial and plant extract showed a significantly higher value of R/S III compared to the control, with SJ treatment being the highest.

From Fig. 2, from the chemical stimulants the significant least value for SVI I was observed for BTH, whereas GB and SA did not show any significant effect although they showed a slight increase in SVI I.

The results of other measured characteristics, namely SG on days 3 and 5, R/S I, GI and SVI III, are presented in Table 2. It was found that there were no significant differences in R/S I in all the treatments as compared to the control. The highest value was found in the ZO treatment of the commercial ginger product. This means that none of the treatment from different sources showed significant differences in root length and/or shoot length compared to control treatment. In case of SG on 3rd day, 97% of the wheat grains germinated in the control, while the grains treated with BTH (83%) and ZO (78%) were significantly lower as compared to the control. The same pattern was observed in SG on 5th day. The GI was significantly lower for BTH (13.4), and ZO (12.7) as compared to the control (15.5). In the case of SVI III, the highest value was observed in RB treatment (1.22 mg) while the lowest value was observed in BTH (0.26 mg) and were statistically higher and lower, respectively, compared to the control (0.79 mg). Furthermore, SVI III for CC (1.13 mg) treatment was also found to be statistically higher compared to control.

**Table 2:** Data of different seed germination characteristics against different treatments. The data is presented as mean ± SD (standard deviation) and compared with control. Different letters following SD represents statistically significant difference at 95% confidence level

Groups	Treatments	R/S I	SG3 (%)	SG5 (%)	GI (unit)	SVI III (mg)
		Mean ± SD				
	Control	1.6 ± 0.31 a	97 ± 0.33 c	97 ± 1.5 c	15.51 ± 0.31 c	0.79 ± 0.1 bd
Chemicals	BTH	1.7 ± 1.22 a	83 ± 8.62 ab	86 ± 6.41ab	13.43 ± 1.22 ab	0.26 ± 0.42 a
	GB	2 ± 0.5 a	91 ± 4 bc	93 ± 2.4 bc	14.73 ± 0.5 bc	0.75 ± 0.12 bc
	SA	1.9 ± 0.6a	95 ± 3.8 c	96 ± 3.7 bc	15.23 ± 0.6 bc	0.86 ± 0.1 bd
Commercial products	CC	2.1 ± 0.6 a	95 ± 3.8 c	96 ± 3.7 bc	15.23 ± 0.6 bc	1.13 ± 0.17 ef
	CL	2 ± 1 a	93 ± 7.22 bc	97 ± 4.8 c	15.07 ± 1 c	0.98 ± 0.11 cdf
	QR	2.1 ± 0.53 a	95 ± 3 c	97 ± 4.1 c	15.33 ± 0.53 c	1 ± 0.14 df
	ZO	6 ± 1.6 a	78 ± 10.16 a	81 ± 9.9 a	12.68 ± 1.6 a	0.67 ± 8.34 b
Wild plant	IG	2.3 ± 0.7 a	94 ± 5 bc	95 ± 3.8 bc	15.08 ± 0.7 bc	0.92 ± 0.25 cde
	RB	2 ± 0.18 a	95 ± 1.82 c	97 ± 0 c	15.33 ± 0.18 c	1.22 ± 0.1 f
	SJ	2.1 ± 0.6 a	95 ± 3.8 c	95 ± 3.8 bc	15.25 ± 0.6 bc	1 ± 0.1 df
	UD	2 ± 0.7 a	95 ± 4.5 c	96 ± 4.34 bc	15.29 ± 0.7 bc	0.98 ± 0.18 cdf

## Discussion

### Chemicals

According to our results, seeds/grains treated with appropriate concentrations of different seed stimulators could enhance the SG and seedling characteristics. The effect of BTH on crops has been studied primarily for protection against different plant pathogens. However, Godard et al. (1999) found that BTH reduced the growth of Billabong cauliflower accession seedlings in a dose dependent manner. The same was also confirmed by Azami-Sardooei et al. (2013), where they found that a BTH concentration greater than 100 mg/L significantly reduced plant Height, flower and fruit numbers in bean and cucumber under pathogen-free conditions, while the highest BTH dose of 1000 mg/L negatively affected tomato vegetative and generative growth. In our study, we also found that the vegetative growth of wheat grains was significantly reduced by applying 1.2 mM of BTH.

Enhancing the SG and the vigour under different adverse environmental conditions is of prime importance in agriculture. Although in this study we did not have any external abiotic stressor. However, some of the chemicals that we investigated have been specifically studied in the presence of different abiotic stressors. One of the chemicals under study is GB. GB has shown promising results in improving SG and seed characteristics. Zhang et al. (2014) performed seed preparation using GB on six turfgrass species in two warm-season grasses under different stress conditions such as drought, salinity, and suboptimal temperature. They found that different turfgrass species showed a difference in sensitivity for different stress conditions. They concluded that the efficacy of GB priming is dependent on three parameters, i.e., the plant, GB concentration, and the stressor. Meanwhile, Ahmed et al. (2019) reported that GB-primed wheat seeds significantly improved SG and seedling characteristics. The authors explained that GB treatment of seeds/grains with appropriate concentration can overcome different inhibitory effects due to water stress by higher antioxidant activity and maintenance of cellular membrane integrity and help the seed/grain survival with good growth. Another possible explanation by Zhang et al. (2022) in their study on tomato, could be that in case of stress, GB might lead to production of stress response-regulatory proteins or downstream production of protective proteins or metabolites. Although there was no biotic or abiotic stress involved in our study, we did not find any significant negative effects of GB treatment on wheat grains. However, we found that GB treatment of wheat grains improved dry root and shoot weight which is calculated for R/S III as seen in Fig. 1.

Another chemical studied extensively for SG and growth in the presence of abiotic stress is salicylic acid (SA). In our study, the application of 0.1 mM SA did not have a significant influence on the characteristics, namely R/S I, SG3, SG5, GI, SVI I and SVI III. However, R/S III increased significantly in SA treatment as compared to control. This means that the dry root weight and the dry shoot weight increased significantly in the presence of SA. Kulak et al. (2021) also reported that the 0.05 and 0.1 mM concentration of SA priming of basil seeds increased not only different seedling characteristics but also other parameters such as relative water content, water potential, quantum yield (Fv/Fm ratio) under well-watered and drought conditions. Our results were similar to those of Dolatabadian et al. (2009) but not under salt stress. They found that, under salt stress, SA significantly increased germination in treated wheat seeds and increased plant growth by increasing cell division in seedlings and roots. SA down-regulates ethylene biosynthesis in seeds, increasing the chances of SG and growth (Ahmed et al. 2020). However, in our study, we did not test this hypothesis. Our results were similar to those of Jadhav and Bhamburdekar (2011) when they treated four different varieties of groundnut with different concentrations of SA. They generally concluded that the 50-ppm concentration of SA increased germination in all the groundnut varieties. Furthermore, they also found that SA had a positive effect on root and shoot growth in 3 varieties, and an antagonistic effect was found in one groundnut variety. It could be argued that such variable effects of SA could be species- or variety-specific.

### Commercial products

The alcoholic extracts of QR, CL were tested on wheat grains only to find no significant increase in R/S I, SG3, SG5, GI and SVI III, except for CC which showed a significant increase in SVI III only, indicating an increase in the dry root and dry shoot weight of wheat seedlings. In the case of ZO, SG3, SG5 and GI was significantly lower as compared to the control, but the highest value of R/S I (6) was observed. R/S III was significantly increased by all commercial products (Fig. 1), while only CC, CL and QR showed a significant increase in SVI I and not ZO (Fig. 2).

These mixed results in the case of CC could be attributed to the two main phenolic compounds such as monoterpenes [carvone (50–80%) and limonene], found in them (Almehemdi and Alsatoori 2019). The allopathic effects of CC on germination and seedling growth of seedlings of wheat, maize, flax, and canary grass under laboratory conditions (Marichali et al. 2014). Contrasting results were obtained compared to ours, as they found that CC leaf extracts of CC significantly inhibited SG and seedling growth characteristics of wheat, maize, flax and canary grass. Such a contrasting result could be attributed to the difference in concentration of these major phenolic compounds found in different parts of the CC plant. Despite the negative effect on SG, Almehemdi and Alsatoori (2019) found that the alcoholic extract of CC fruits was effective killers of field dodder (*Cuscuta campestris*), a flowering parasitic plant. Perhaps such an application of different caraway parts that can kill unwanted and invasive plants could open a new opportunity for further research.

CL (Turmeric), a traditional spice used in cooking, has also been explored for its anti-inflammatory, antimicrobial, and antioxidant effects (Nobre et al. 2022). Silver nanoparticles mediated by rhizome extract of turmeric was tested for its antifungal effect against *Rhizoctonia solani* in rice (Chintala et al. 2023). They found not only that it displayed a great antifungal efficacy, but also enhanced rice seed characteristics such as germination, root length, and shoot length. Our result also showed that although not significant, but there was an increase in the R/S I. Kim et al. (2021) further tested different concentrations (1, 3, 5%) of turmeric extracts on nutritional and antioxidant activity of germinated Korean brown rice. They found that in general, 1 and 3% could significantly enhance the nutritional and functional value of Korean germinated brown rice. In our study, we did not carry out such an experiment, but it certainly shows that turmeric extract does have a positive effect. This theory should be tested in wheat and other crops in the future. In contrast to the enhancement of germinating properties that we found in our results, Akter et al. (2018) found that methanolic extracts of two CL cultivars (Ryudai gold and Okinawa ukon) inhibited the SG of radish, cress, lettuce and *Bidens pilosa*. However, in this study, the negative effect of turmeric varieties on *B. pilosa* seeds is important because *B. pilosa* is a weed species that is widely found in the world and is a host and vector of some disastrous parasites such as *Meloidogyne* species. This inhibitory effect of turmeric needs further careful attention and research to eliminate weed species, although Akter et al. (2018) found that dihydrobisdemethoxycurcumin, bisdemethoxycurcumin, demethoxycurcumin and curcumin were responsible for the inhibitory effect on SG.

In this study, we did not observe any negative effects of QR on any of the characteristics of wheat grains. Based on our study, we can say that QR extracts could be a potential seed stimulant for wheat grain germination. Ahn et al. (2021), found that 45 days of oak tree sawdust fermentation showed the heaviest biomass weight, longest true leaf and hypocotyl, resulting in the highest total vigour index in peanuts. However, the same was not the case with Alrababah et al. (2009), where they tested the effects of *Pinus halepensis* and *Quercus coccifera* on wheat, barley, lentil, chickpea and faba bean. They found that allelopathic effects of both treatments, that is, *P. halepensis* and *Q. coccifera* reduced crops SG of all the tested with the maximum germination of 75% achieved only in the faba bean. Tahir et al. (2022) tested oak leaf extract, soil containing oak leaf powder, and biofertilizer on two genotypes of tomato under water stress condition. They found that under drought stress all treatments significantly reduced shoot length, total fruit weight per plant, relative water content, and total chlorophyll content compared to control. However, the fresh weight and root dry weight of the roots of the genotypes increased compared to control. Garnett et al. (2004) tested water extracts of three species of leaf litter, namely, pitch pine, huckle berry and white oak, in pitch pine SG. They found that although none of the treatments influenced SG, higher concentrations significantly reduced seedling growth, indicating phytotoxic effects of these species. There are several theories that have been published to understand the negative effects of extracts made from *Quercus* species on SG. The proposed theories were summarised by Alrababah et al. (2009), where researchers McPherson and Thompson in 1972 proposed physio-mechanical inhibition, resource competition or allelopathy as possible reasons for phytotoxic effects; Gliessman in 1978 and Richardson and Williamson in 1988 proposed toxins present in green and freshly fallen leaves of oak that leads to toxic effect thereby reducing SG. Halvorson et al. (2017) found that the extracts of yellow poplar, red maple, and white oak reduced the germination of alfalfa, red and white clover, crabgrass, orchardgrass, and tall fescue. However, when seeds treated with these extracts were washed with water, germination of all the tested resumed, indicating that inhibition could be due to the osmotic effects of extracts or water-soluble allelopathic compounds.

Ginger is another spice that has been traditionally used for culinary purposes and is also known to have antimicrobial properties and allelopathic effects. In our study, we found that ginger significantly inhibited wheat grain germination and germination index but had the highest R/S I and significantly increased R/S III, indicating positive effect on dry root weight and dry shoot weight. Han et al. (2008), who tested rhizome, stem, and leaf aqueous extracts of ginger at different concentrations on SG and early seedling growth of soybean and chive, observed similar results with respect to low germination. They found that all concentrations inhibited SG, seedling growth, water uptake, and lipase activity. Our results were concurrent with those of Ibáñez and Blázquez (2019) who tested the effect of turmeric and ginger essential oils against 5 weed species namely *Portulaca oleracea*, *Lolium multiflorum*, *Echinochloa crus-galli*, *Cortaderia selloana*, and *Nicotiana glauca* and 3 crops namely tomato, cucumber, and rice. They found that ginger essential oil significantly inhibited the SG of *P. oleracea*, *L. multiflorum*, and *C. selloana* at highest concentration. In tomato, they found that 1 µL/mL of ginger essential oil did not affect germination but significantly inhibited hypocotyl and radicle development. Meanwhile, no such negative effects were observed in rice and cucumber. Such results can be attributed to different constituents present in ginger, especially higher levels of monoterpenes (1.8-cineole) (Miranda et al. 2015). Various phenolic metabolites such as gingerols, shogaols, paradols, zingerones, gingerdiones and diarylheptanoids are identified but not tested in agronomy (Zorrilla et al. 2024). These metabolites alone or in combination could be one of the probable reasons for such negative effect on seed germination of wheat. Our results are in agreement with Gemoto et al. (2022), where they found that the decrease in the percentage of germination was directly proportional increase in the concentration of ginger leaf extract in mung beans (*Vigna radiata*), while the germination rate was significantly low only at the highest concentration of the extract.

### Wild plants

IG alcohol leaf extracts did not show any significant decreases in in R/S I, or SG3, nor SG5 nor GI. However, R/S III and SVI I were significantly higher, indicating higher values of dry root weight and dry shoot weight and higher seedling length respectively (Fig. 1, Fig. 2, Table 2). Our results were contrasting to the study of Baležentienė (2018) who tested the phenolic content in IG on wheat and rape SG only to find that the phenolic content significantly inhibited germination and seedling growth in both the tested species with the strongest inhibition found in rape SG. Furthermore, hypocotyl and radicle exhibited stronger response to IG extracts as compared to wheat, resulting in a significant decrease, which could be attributed to different seed coat anatomy and its permeability. Mynard and Vilas (2023) tested the impact of IG on three native plants, namely *Trifolium pratense, Linum grandiflora,* and *Silene dioica*. They found that seedlings and residues (above-ground plant matter) of IG reduced chlorophyll content and growth (measured as above-ground dry mass) in the three native species. However, the presence of IG residues had a positive effect on the above-ground dry mass of *S. dioica*, which we found in our results with R/S III and SVI I in wheat. The IG shoot extract is more potent as compared to the root extract, as the shoot extract exhibited significantly higher inhibitory effect on SG of two native species, namely *Hieracium murorum* and the growth of *Scrophularia nodosa* and mycelium of three ectomycorrhiza fungi as compared to the root extract but only in higher concentration (Ruckli et al. 2014). They found that it is due to the presence of the 2-methoxy-1,4-naphthoquinone (2-MNQ) compound in the root and leaf extract. In our study, our main objective was to test the effect of IG extract on seed germination and thus we did not perform the chemical analysis of the extract.

RB is an invasive plant species that has the potential to hinder the growth of native plants through allelopathy, making the native plants fight for their survival (Murrell et al. 2011). Soil substrate contaminated with three knotweed species, namely *R. japonica* (Japanese knotweed), RB (Bohemian knotweed), and *Reynoutria sachalinensis* (Giant knotweed) were tested for germination properties in two crop plants (*Leucosinapis alba*, *Brassica napus*) and two weed plant species (*Chenopodium album* agg., *Echinochloa crus*-*galli*) (Šerá 2012). The three knotweed species significantly reduce germination of *L. alba* seeds (< 10%). In *B. napus*, only RB did not have a significant effect on SG. Regarding the two weed species under study, none of the knotweed species exerted a significant negative effect on them. We obtained a contrasting result, as RB did not exert any significant negative effects on any of the parameters. In fact, a significant increase in SVI I, SVI III, and R/S III indicates that RB exerted a positive stimulation effect that resulted in an increase in below and above ground mass (Fig. 1, Fig. 2, Table 2). Similar results with RB positively influencing parameters such as seedling vigor indices I and II, R/S dry weight, shoot and seedling lengths, root, shoot, and seedling dry weights were reported by Šerá et al. (2024). Our results coincide with Parepa et al. (2012) as they found that RB did not suppress germination or early growth of nine different native plants. They concluded that the allelopathic effect of RB is on the growth of the plant rather than SG and that the persistence of these allelochemicals in the soil may not have a significant problem in habitat restoration. Šoln et al. (2022) discovered that the extracts of knotweeds of radish seeds and root growth were derived from tests of knotweeds, containing plant chemicals such as resveratrol, emodin, (–)-picatechin, (–)-catechin, piceid, and resveratroloside. But they reported that resveratrol in *R x bohemica* had greater concentrations than in *R. japonica*, thereby inhibiting germination of radish seeds. Our study did not quantify the phytochemical content of RB, but based on our results, we hypothesized that the resveratrol content in our extract of RB might be much lower, thereby having a negative impact on the germination of wheat seeds and seed growth.

The use of SJ extract for seed priming or for testing SG is not usual. In this article, we tested the alcoholic extract of SJ on wheat grain stimulation. No significant negative effect was observed on any of the characteristics of the wheat grains studied. In fact, the R/S III value was found to be highest in the SJ treatment, indicating that the SJ extract had a positive effect on the below-ground biomass. Furthermore, SJ extracts also enhanced SVI I index in wheat grains. No previous data are available to compare the effects of SJ extract on agricultural crop seed/grain characteristics. However, *Acacia cyanophylla* which is another tree species in the *Fabaceae* family; were tested on two crops (*Triticum aestivum* and *Lactuca sativa*) and two species of weeds (*Peganum harmala* and *Silybum marianum*) (Ayab et al. 2013). They found that the extracts showed phytotoxicity against all the species except wheat. But overall, they concluded that *A. cyanophylla* contains allelochemicals that can be used as a bioherbicide. Kamel and Hammad (2015) obtained similar results as they found that wheat was more tolerant than canola to the aqueous extracts of *Acacia saligna*. From the above studies, we can say that wheat grains are tolerant to the different allelochemicals found in *Acacia* sp. but the allelochemicals present in SJ need to be tested to understand the exact reasons behind the stimulation of wheat grains.

From our study, we found that alcoholic UD extracts significantly enhanced the R/S III and SVI I index with no evident negative effects on other characteristics of wheat grains (Fig. 1, Fig. 2). Our results agree with the study by Maričić et al. (2021) where they tested short-term and long-term extract on *Phaseolus vulgaris* under field conditions. They found that the short-term extract with foliar application was as efficient long-term extract with soil irrigation, showing a positive effect on green bean vegetative parameters and iron accumulation in the leaves. This was predominantly due to the nutrients and other components present in the extract rather than nitrogen fixing ability of nettle extract (Maričić et al. 2022). On the contrary, recently Kumar et al. (2025) found that both leaf root extracts from UD have a very significant negative effect on *Glycine max* (Soybean) and *Linum usitatissimum* (Flax). This variable effect of UD could be attributed to the inter-species sensitivity with UD. Meanwhile, Quratul-Ain et al. (2023) discovered that the phytotoxicity of the UD stem extract on *Zea mays* and *Cassia sophera* is directly associated with the concentration of the stem extract. They identified 47 volatile compounds from ethanolic stem extract of UD. It is possible that some of these compounds could be present in our extract, but in lower concentrations as we could not find any significant negative effect on wheat seed parameters. However, we recommend further testing and investigating the different volatile compounds present and their individual and combined effect on wheat seed germination and seedling growth.

## Conclusion

Different plant stimulants were selected based on our previous literature and additional sources and tested on wheat grain germination and seedling characteristics under laboratory conditions. Germination characteristics such as seed germination (SG), germination index (GI) and initial seedling growth characteristics such as root: shoot ratios (R/S I and III) and seedling vitality indexes I (SVI I, III) were measured. Benzothiadiazole and ginger extract showed significantly less SG and GI compared to the control sample. No significant effect on R/S I was observed, but the highest value was observed in ginger extract. The SVI III index was significantly low for benzothiadiazole while significantly higher for turmeric and bohemian knotweed extracts. In the case of R/S III, except benzothiadiazole, all treatments showed significant increases. For the SVI I index, benzothiadiazole showed the least significant value, while glycine betaine, salicylic acid and ginger extract showed no significant changes. All other treatments significantly improved the SVI I index. Although we did not perform the chemical tests to understand which compounds were involved, it opens a new area of research for us to understand their effect in detail and in the future, if possible, commercially produce them for testing and field trials.

## Data Availability

All data generated and analysed during this study are available from the corresponding author upon reasonable request.
